# Medical Student Evaluation With a Serious Game Compared to Multiple Choice Questions Assessment

**DOI:** 10.2196/games.7033

**Published:** 2017-05-16

**Authors:** Julien Adjedj, Gregory Ducrocq, Claire Bouleti, Louise Reinhart, Eleonora Fabbro, Yedid Elbez, Quentin Fischer, Antoine Tesniere, Laurent Feldman, Olivier Varenne

**Affiliations:** ^1^ AP-HP, Hôpital Cochin Cardiology Paris France; ^2^ Université Paris Descartes Paris France; ^3^ iLUMENS Department of Simulation University of Sorbonne Paris Cité Paris France; ^4^ Université Paris Diderot Paris France; ^5^ AP-HP, Hôpital Bichat Cardiology Université Paris Diderot Paris France; ^6^ FACT, French Alliance for Cardiovascular Trials, DHU FIRE Cardiology department of Bichat hospital Université Paris Diderot Paris France; ^7^ URC-Est AP-HP Paris France; ^8^ AP-HP, Hôpital Cochin Anesthesiology Paris France

**Keywords:** serious game, multiple choice questions, medical student, student evaluation

## Abstract

**Background:**

The gold standard for evaluating medical students’ knowledge is by multiple choice question (MCQs) tests: an objective and effective means of restituting book-based knowledge. However, concerns have been raised regarding their effectiveness to evaluate global medical skills. Furthermore, MCQs of unequal difficulty can generate frustration and may also lead to a sizable proportion of close results with low score variability. Serious games (SG) have recently been introduced to better evaluate students’ medical skills.

**Objectives:**

The study aimed to compare MCQs with SG for medical student evaluation.

**Methods:**

We designed a cross-over randomized study including volunteer medical students from two medical schools in Paris (France) from January to September 2016. The students were randomized into two groups and evaluated either by the SG first and then the MCQs, or vice-versa, for a cardiology clinical case. The primary endpoint was score variability evaluated by variance comparison. Secondary endpoints were differences in and correlation between the MCQ and SG results, and student satisfaction.

**Results:**

A total of 68 medical students were included. The score variability was significantly higher in the SG group (σ^2^ =265.4) than the MCQs group (σ^2^=140.2; *P*=.009). The mean score was significantly lower for the SG than the MCQs at 66.1 (SD 16.3) and 75.7 (SD 11.8) points out of 100, respectively (*P*<.001). No correlation was found between the two test results (R^2^=0.04, *P*=.58). The self-reported satisfaction was significantly higher for SG (*P*<.001).

**Conclusions:**

Our study suggests that SGs are more effective in terms of score variability than MCQs. In addition, they are associated with a higher student satisfaction rate. SGs could represent a new evaluation modality for medical students.

## Introduction

Student evaluation is one of the most important components of a medical educational program and is used for training and for validating degrees and career options. If handled well, it can improve student motivation for learning and provide educators useful feedback. Medical education cannot be limited to book-based knowledge which is defined as the ability to provide an answer from medical literature [1,2]. It also comprises developing medical skills such as the ability to act to obtain medical data and provide good care to patients [3]. Therefore, given the importance of questioning and the deductive process required to reach the right diagnosis and prescribe the right treatment, proper evaluation modalities are needed based on both book-based knowledge and diagnostic skills. High score variability, defined as the highest score in points obtained between students, is also mandatory to allow representative classification and fair career access based on test results in large student populations. On the other hand, the evaluation modality should also allow for an objective, fast, and inexpensive correction. As such, multiple choice questions (MCQs) are currently the most frequently used modality. Medical serious games (SG), based on virtual reality, are emerging as an alternative way of evaluating medical education [4]. However, they have not yet been evaluated in terms of score variability. In this study, we sought to evaluate medical students’ test results with an SG compared with MCQs in terms of score variability, score difference, correlation between scores in MCQs and SG, student satisfaction, and finally whether SGs could be of use to learn and evaluate medical skills for medical students.

## Methods

### Study Design

From January to September 2016, we included all volunteer medical students with previous cardiology validation in two medical schools (University Paris Descartes, Paris, France and University Denis Diderot, Paris, France). Students were randomized in a cross-over design between two groups to avoid order bias. Group 1 started with evaluation by SG and finished with evaluation by MCQs and group 2 performed alternatively. The tests were performed in the examination centers of both medical schools. Both tests lasted 30 minutes and the tests were performed consecutively. The study was approved by the educational committee of both institutions. All students gave their informed consent before inclusion.

### Serious Game

**Figure 1:**
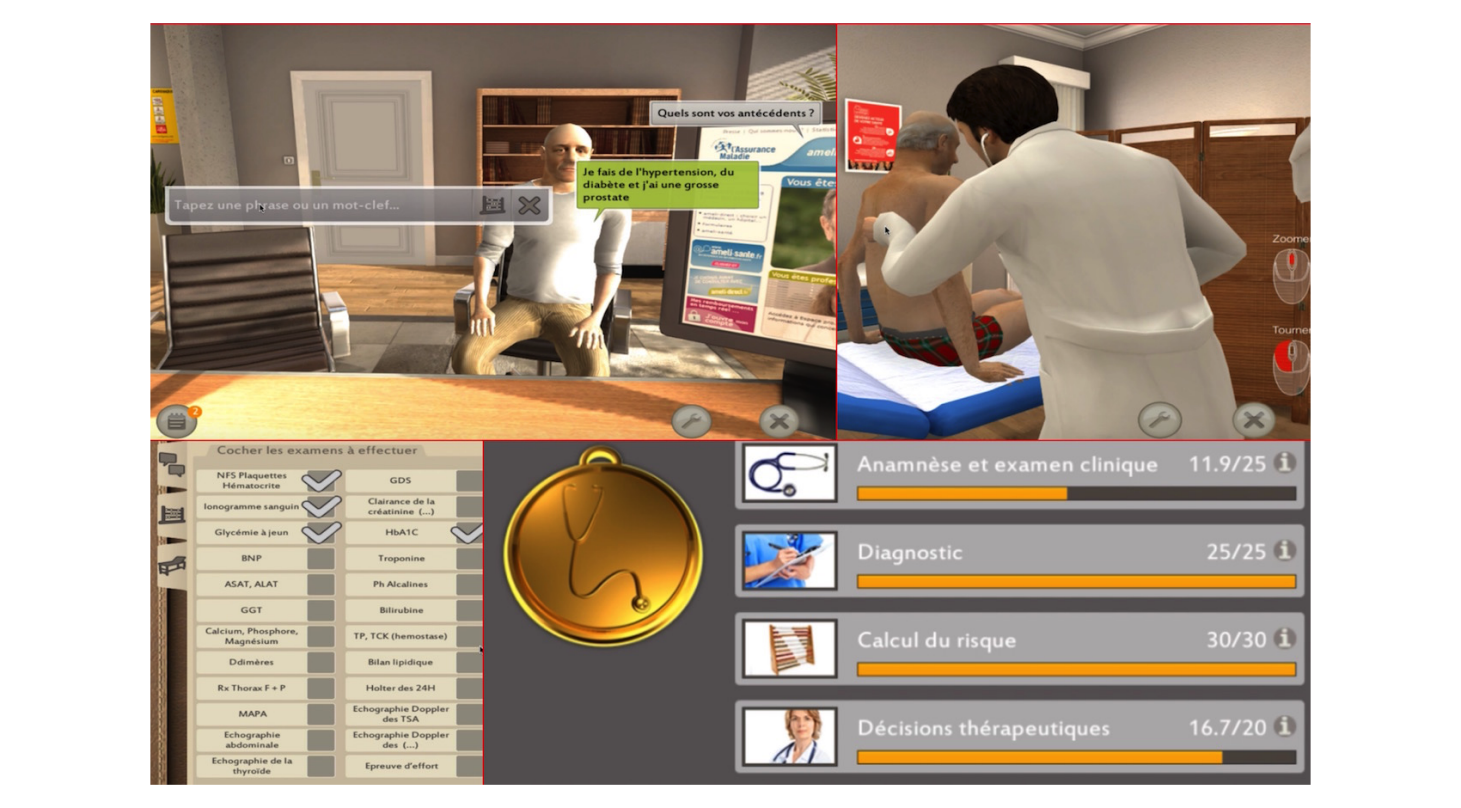
Serious game illustration (in French).

We used a clinical case from an SG (Medusims, Paris, France and iLUMENS, Medical Simulation Department, Université Sorbonne Paris Cité, Paris, France). The SG focuses on the management of atrial fibrillation. It represents a cardiologist and a patient within a free tridimensional (3D) environment within a medical office, and is available on computers and tablets (Figure 1). Students play the role of the cardiologist and ask the patients questions using key words, perform a complete clinical examination with electrocardiogram, and require the prescription of additional tests and medical treatment. Points are awarded if the student asks the patient a correct question or performs the appropriate physical examination. There are no negative points due to wrong answers. Besides the free conduct of the clinical questioning, pop-up questions also arise in the SG during electrocardiogram interpretation, risk score calculation and the potential medical treatment in form of MCQs. Points are also awarded for correct answers to these pop-up questions. An automatic and precise correction is given to the student at the end of the game. Results are expressed out of a total of 100 points divided into four subcategories: clinical examination out of 25 points, diagnosis out of 25, risk score calculation out of 30 and medical decision out of 20 (Figure 1).

### Multiple Choice Questions

We built an online MCQ test of 15 questions based on the SG clinical case with the same clinical and electrographic presentation. Each MCQ presented five possible answers. The student scored full points if all the selected answers were correct, 50% if one answer was incorrect, and 20% if two answers were incorrect. No points were awarded if three or more answers were incorrect. The correction was aligned to the SG correction giving a final score out of 100 points. A translated version of the MCQ test is available in the Multimedia Appendix 1.

### Satisfaction and Student Description Questionnaires

**Figure 2:**
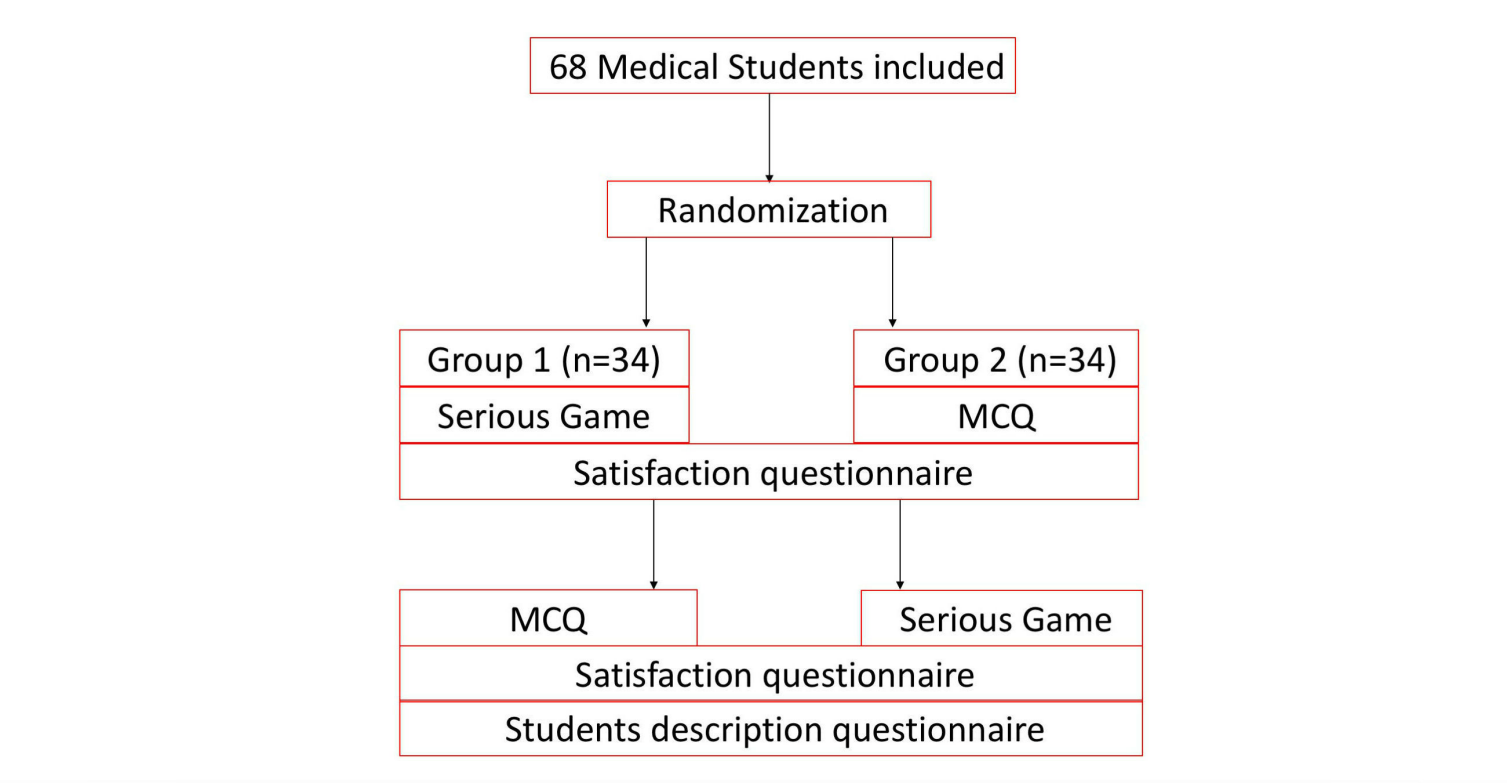
Flowchart.

Questionnaires to record student characteristics and satisfaction were designed by a psychologist from the Medical Simulation Department of University Paris Descartes (iLUMENS, Paris, France). The student satisfaction questionnaire was filled in immediately after each evaluation using website. The student characteristics questionnaire was filled in online at the end of the study protocol to assess the medical degree and whether the student played video games regularly at the time of the study (gamers) or not (non-gamers; Figure 2).

### Objectives and Endpoints

The primary objective of the study was to compare the students’ scores at the MCQs and SG tests. The related primary endpoint was therefore the score variability calculated as a variance for each test. The secondary endpoints were student satisfaction with semiquantitative questions expressed from 0 (no, not at all) to 5 (yes, entirely) and the correlation between the test results. Subgroup analysis was performed for SG results between gamers and non-gamers.

### Statistical Analysis

Summary descriptive statistics are reported as mean and standard deviation, median (inter quartile range), or counts (%), as appropriate. We used the *t* test and Mann-Whitney test to evaluate the difference of continuous variables as appropriate. Fisher exact test was used for variance comparison. For score differences, the paired *t* test was used. Finally, the correlation coefficient was calculated between the results of SG and MCQs using the Pearson R^2^ correlation test. All analyses were performed with SPSS 21.0 (IBM Inc), R 3.3.1 (R Project for Statistical Computing) and Prism GraphPad 7.0 (GraphPad Software Inc).

## Results

### Main Student Characteristics

A total of 68 medical students were included (34 in each group), of which 29 were male (43%) and the mean age was 23(SD 1) years. Students were in their 5th [4-6] year of medical school. All the students owned a cellphone and a personal computer, and subscribed to an Internet connection; 31 (46%) owned a tablet and 21 (31%) a video game console. A past experience of video games was reported by 60 (88%) of students and 22 (32%) were currently playing video games for an average duration of 1.6(SD 3.0) hours per week. The main characteristics of the population according to the allocated group of randomization are detailed in Table 1. There were no significant differences in student characteristics between groups 1 and 2.

**Table 1:** Student characteristics.

Student description N=68	Overall	Group 1 n=34	Group 2 n=34	Comparison between groups 1 and 2 (*P* value)
Sex (male), n (%)	29 (43)	17 (50)	12 (35)	.22
Age in years, mean (SD)	23 (1)	23 (1)	23 (1)	.26
Year of medical school, mean (SD)	4.7 (1.0)	4.7 (0.8)	5.1 (0.9)	.08
Cardiology internship within the past 12 months, n (%)	34 (50)	16 (47)	18 (53)	.74
Owns a cell phone with Internet connection and social network account, n (%)	67 (99)	33 (97)	34 (100)	>.99
Owns a tablet, n (%)	31 (46)	13 (39)	18 (54)	.20
Owns a computer with Internet connection possession, n (%)	68 (100)	34 (100)	34 (100)	>.99
Owns a video game console, n (%)	21 (31)	14 (42)	7 (20)	.07
Past video game experience, n (%)	60 (88)	28 (83)	32 (94)	.26
Age in years at first video game experience, mean (SD)	9 (3)	9 (3)	9 (3)	.51
Currently playing video games, n (%)	22 (32)	14 (40)	8 (26)	.31
Hours of video game per week, mean (SD)	1.6 (3.0)	1.9 (3.7)	1.3 (2.1)	.65

### Test Results

**Figure 3:**
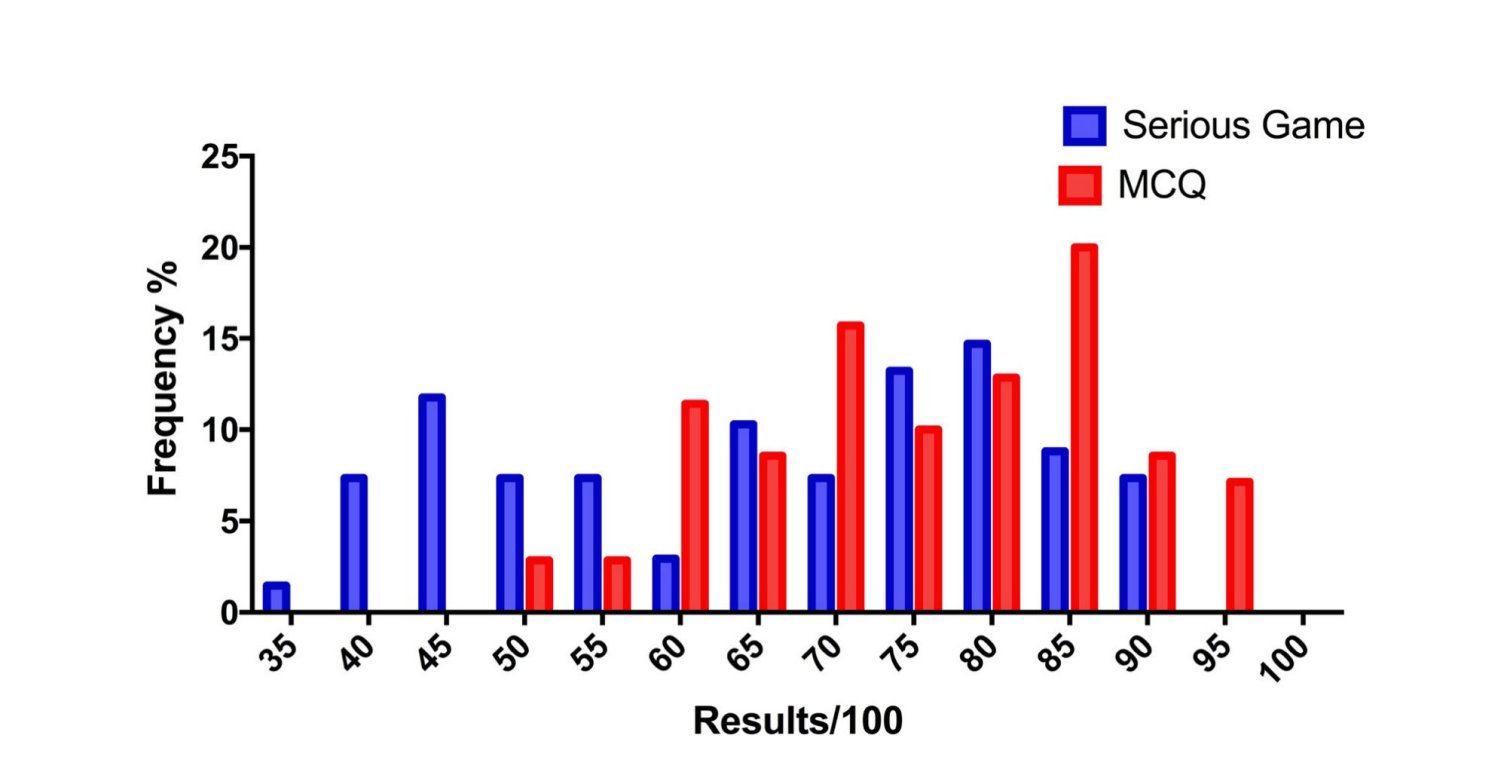
Result’s histogram.

**Figure 4:**
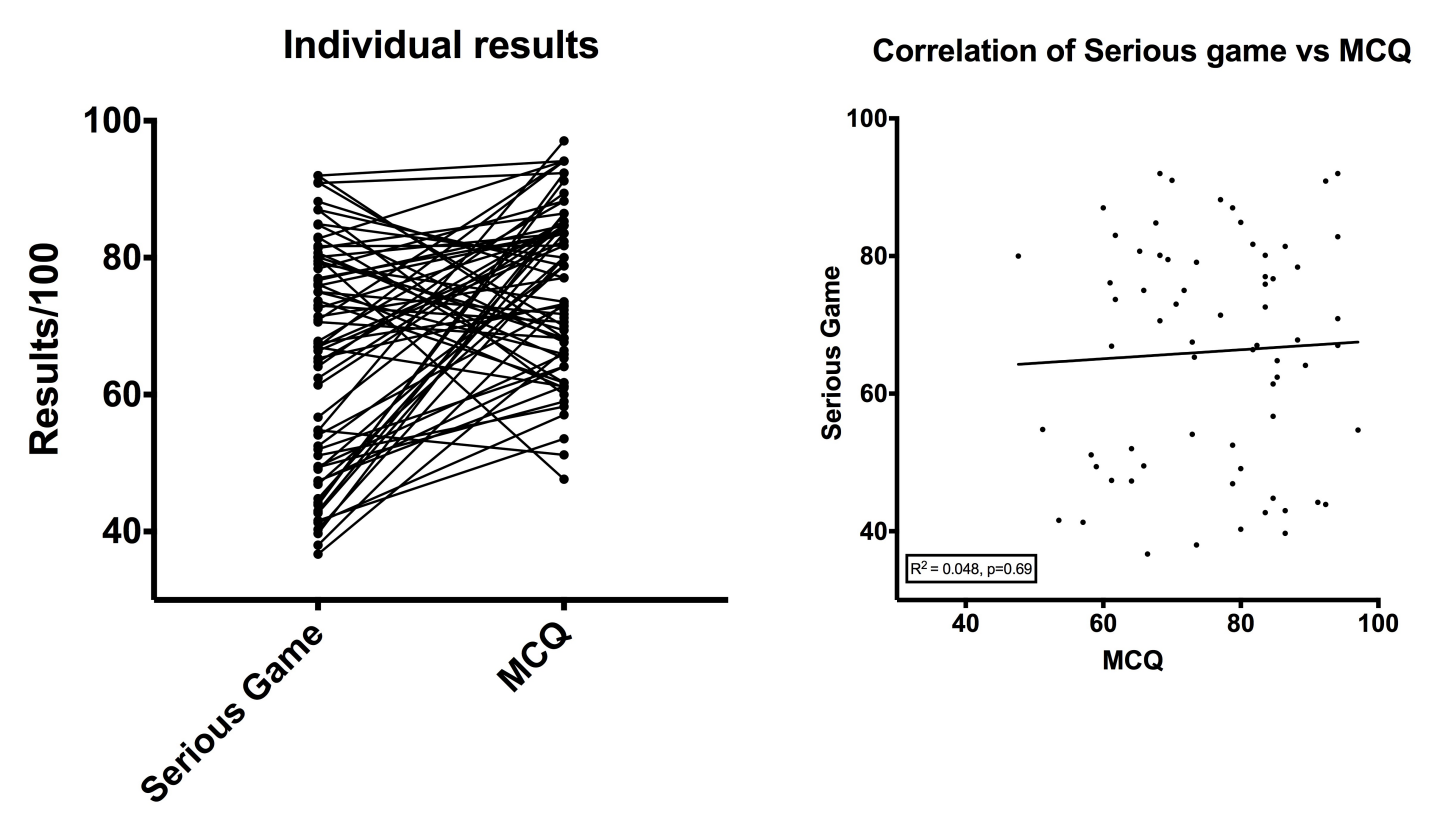
Individual test results in the left panel (A) and correlation coefficient in the right panel (B).

The score variability expressed as variance of the students’ results was significantly higher in the SG group (σ^2^=265.4) compared with MCQs group (σ^2^=140.2; *P*=.009), as illustrated in Figure 3. The overall results for each test were significantly lower for SG (mean 66.1, SD 16.3 points) compared with MCQs (mean 75.7, SD 11.8 points; *P*<.001). For both the SG and MCQs, the results were better when the student had already performed the other test before: 62.0 (SD 15.2) points for the SG when it was performed first versus 70.2 (SD 16.5) points when performed second (*P*=.02); 67.4 (SD 8.9) points for the MCQs when it was performed first versus 83.9 (SD 8.1) points when performed second (*P*<.001). No correlation was found between the results of the two tests: R^2^=0.048 (*P*=.58; Figure 4). No significant difference was observed between gamers (22/68; 32%) and non-gamers (46/68; 68%) for SG results, respectively 65.8 (SD 13.3) versus 66.2 (SD 17.4) points (*P*=.71).

**Table 2:** Satisfaction questionnaire: results are expressed as mean (SD) of numeric ordinal variable from 1 (no, not at all) to 5 (yes, entirely).

Questions	Serious game	Multiple choice questions	*P* value
Did you encounter difficulties to answer the questions?	2.18 (1.14)	2.21 (1.14)	.89
Were you able to concentrate while answering the questions?	3.93 (0.99)	3.71 (1.06)	.15
Do you think that this test is close to clinical reality?	4.21 (0.75)	2.68 (0.88)	<.001
Did you find this test stressful?	2.51 (1.05)	2.30 (1.17)	.24
Did you understand the goal of the test?	4.24 (0.75)	3.97 (0.94)	.10
Do you consider that this kind of test represents a proper evaluation?	3.91 (0.87)	3.04 (1.02)	<.001
Are you satisfied with your test performance?	3.05 (1.09)	3.22 (0.98)	.41
Did you think that your knowledge progressed after this test?	3.56 (1.09)	2.42 (0.99)	<.001
Are you satisfied with this type of evaluation?	3.88 (1.42)	2.98 (1.53)	<.001

**Table 3:** Assessment of serious games as a tool to learn medicine. Results are expressed as mean (SD) of numeric ordinal variable from 1 (no, not at all) to 5 (yes, entirely).

Assessment of serious games as a tool to learn medicine	Serious game
Educational quality	4.86 (0.35)
Feeling of connection or attachment to the serious game	3.60 (1.19)
Possibility of playing with other students	3.26 (1.18)
Possibility of comparing results with other students	3.44 (1.33)
Fun	3.37 (1.16)
Original, innovative or new	3.90 (0.98)
Possibility to adapt level of difficulty	4.36 (0.68)
Availability on smartphone	4.00 (1.07)

### Satisfaction Analysis

The satisfaction questionnaires showed a significantly higher overall self-reported satisfaction for the SG compared with the MCQ test. Students reported that the SG was closer to clinical practice, represented a proper evaluation and that they felt to have learned more with the SG than with MCQs, thus representing a better evaluation modality (*P*<.001 for all). Conversely, students did not experience significant differences between the two test modalities in terms of understanding, answering the questions, performance satisfaction or stress generated by the test (*P* value non-significant for all; Table 2).

### Serious Games as a Tool to Learn and Evaluate Medical Skills

The questionnaire was also designed to evaluate whether students thought that SGs could be an interesting tool to learn and evaluate medical skills. Most of the students thought that it could be. The highest ranking points (>4) were educational quality, the possibility of adapting the level of difficulty of an SG and the availability on smart phone (Table 3).

## Discussion

### Principal Findings

This study is the first to compare an SG to MCQs in terms of score variability for medical students. This study demonstrates that the SG was associated with a higher score variability and lower mean score compared with MCQs. Moreover, the SG was associated with significantly higher student satisfaction compared with MCQs. Most medical student evaluation to date is based on MCQ tests which are performed on a large student population. Student grading might therefore be difficult with a sizable proportion of students scoring the same and limited score variability between them. We believe that tests evaluating a large population of medical students should include overall results variability and be of high student satisfaction. For these reasons, we sought to evaluate medical students with a simulation based on an SG compared to MCQs. MCQs evaluate medical knowledge by the means of closed questions, but medical skills and competence are better assessed by on site (bedside) evaluation or simulation [5-8]. While the lines between SGs and simulations are somewhat blurred, an SG represents a virtual world. It is generally played alone with completion based on a score while a simulation is performed on site with an instructor or in a group without score. Several studies have reported higher student satisfaction with simulation compared to MCQs [9,10]. SGs have several potential advantages over simulations to evaluate medical students. Simulation programs are expensive and time-consuming which limits access. Although production of medical SGs is expensive, once the 3D environments have been created it is less expensive to build new SGs using the same environment. Furthermore, they can be easily shared throughout a large medical student community. SGs also increase the realism of clinical situations [11] and evaluate both medical knowledge and competences via simulation and unguided actions in a 3D environment [12]. Importantly, we believe that SGs exemplify the human desire to play and to master challenges. Besides their potential use as an evaluation tool, SGs might also be an interesting way to train and to teach medicine. The student is drawn “into the game” making medical knowledge and skills more easily transmitted and retained. This use of SGs as a learning tool is supported by our study: students gave a high score for the educational quality of SGs. They particularly appreciated the possibility of accessing SGs with their smartphone and the personalized difficulty feature Finally, SGs also offer the advantage of self-assessment.

Medical education encompasses both medical knowledge and reasoning skills. Although it is simple to develop MCQs to test medical knowledge, it becomes much more challenging to evaluate reasoning skills and global medical skills with MCQs. Interestingly, our study did not find any correlation between the two sets of test results, suggesting that success in MCQs does not predict success in SGs and vice versa. This finding might suggest that good results in an SG are different from pure medical knowledge evaluation and that medical skills might increase result variability since the medical knowledge tested were similar in both tests. If SGs are considered to be closer to medical practice, this finding questions the effectiveness of MCQs in evaluating medical students [10,13]. This finding possibly suggests that the tests evaluate different reasoning skills and abilities to perform.

We acknowledge several limitations in our study. Our study compared two different test modalities evaluating a relatively small number of medical students in managing a cardiology clinical case. Therefore, further studies are needed to confirm our findings in larger student populations and in other medical fields. Although we found an order bias in our study—the second test was associated with better results because of similar questions, retention of the students’ answers, and indirect access to the corrections—we believe that randomization in two similar groups allowed us to draw reliable conclusions. As specific questionnaires were designed for this study, no pretest was conducted. Nevertheless, we believe that the questionnaires are valid, since each student acted as is his own control in this study, interpreting the questions in the same way when evaluating two different test modalities. Finally, our sample consisted of volunteer students and we cannot rule out the fact that they might have a particular interest in SGs. This might also limit the generalization of our conclusions to the whole population of medical students.

### Conclusions

SGs potentially represent a new evaluation modality for medical students. Our study suggests that they are more effective in grading medical students with a higher variability of performance. In addition, SGs seem to be associated with higher student satisfaction compared to MCQs.

## Appendix

Multimedia Appendix 1 Multiple choice questions test translated in English.

## Abbreviations

MCQs: Multiple choice questions
SG: Serious games
SD: Standard Deviation

## References

[1] Liu Q, Peng W, Zhang F, Hu R, Li Y, Yan W (2016). The effectiveness of blended learning in health professions: systematic review and meta-analysis. J Med Internet Res.

[2] Menet A, Assez N, Lacroix D (2015). Cross analysis of knowledge and learning methods followed by French residents in cardiology. Arch Cardiovasc Dis.

[3] Ryall T, Judd BK, Gordon CJ (2016). Simulation-based assessments in health professional education: a systematic review. J Multidiscip Healthc.

[4] Graafland M, Dankbaar M, Mert A, Lagro J, De Wit-Zuurendonk L, Schuit S, Schaafstal A, Schijven M (2014). How to systematically assess serious games applied to health care. JMIR Serious Games.

[5] Gordon Ja, Oriol Ne, Cooper Jb (2004). Bringing good teaching cases “to life”: a simulator-based medical education service. Acad Med Jan.

[6] Scouller K (1998). The influence of assessment method on students' learning approaches: multiple choice question examination versus assignment essay. Higher Education.

[7] Palmer EJ, Devitt PG (2007). Assessment of higher order cognitive skills in undergraduate education: modified essay or multiple choice questions? Research paper. BMC Med Educ.

[8] Scalese RJ, Obeso VT, Issenberg SB (2008). Simulation technology for skills training and competency assessment in medical education. J Gen Intern Med.

[9] Wahlgren C, Edelbring S, Fors U, Hindbeck H, Ståhle M (2006). Evaluation of an interactive case simulation system in dermatology and venereology for medical students. BMC Med Educ.

[10] Solymos O, O'Kelly P, Walshe CM (2015). Pilot study comparing simulation-based and didactic lecture-based critical care teaching for final-year medical students. BMC Anesthesiol.

[11] Johnsen HM, Fossum M, Vivekananda-Schmidt P, Fruhling A, Slettebø Å (2016). Teaching clinical reasoning and decision-making skills to nursing students: design, development, and usability evaluation of a serious game. Int J Med Inform.

[12] Virvou M, Katsionis G, Manos K (2006). Combining software games with education: evaluation of its educational effectiveness. J Educ Techno Soc.

[13] Duque G, Fung S, Mallet L, Posel N, Fleiszer D (2008). Learning while having fun: the use of video gaming to teach geriatric house calls to medical students. J Am Geriatr Soc.

