# Supplementary material for: Medical Student Evaluation With a Serious Game Compared to Multiple Choice Questions Assessment
Source: JMIR Serious Games. 2017 May 16;5(2):e11. doi: 10.2196/games.7033 (PMC5449650; doi:10.2196/games.7033)
Supplement: Multimedia Appendix 1 [file games_v5i2e11_app1.pdf]

Appendix 1: Multiple choice questions test translated in English.

The online version show only one question per page the student cannot go to the next question without validating his answer, after validation it is not possible to go back.

A 70 years old male with past history of prostate adenoma consult for dyspnea NYHA class II.
Risk factors diabetes and hypertension.
No smoker, no alcohol abuse.

Q1: What questions do you ask for his complain?

A. Did the patient had discomfort?

B. What are his medication?

C. Did he have dyslipidemia?

D. Did he felt palpitations?

E. Did he had chest pain?

The patient take statin, oral antidiabetic medication and angiotensin-converting enzyme inhibitors. The patient felt palpitations and had dyspnea.

Q2: What are the essential questions to ask to this patient?

A. Describe palpitation

B. Describe dyspnea

C. Did the patient experience loss of consciousness?

D. Did the patient perform regular physical exercise?

E. Did the patient experience headache

Q3: During physical examination, which are the examinations to do in this patient?

A. Blood pressure

C. Neurologic reflex

D. Cardiac auscultation

D. Pulmonary auscultation

E. Radial pulse palpation

The patient had high blood pressure 162/90 mmHg. Radial pulse palpation showed irregular tachycardia at 140/min. Cardiac and pulmonary auscultations are normal.

Q4: What test could you perform?

A. EKG

B. Urinary dipstick test

C. Trans thoracic cardiac echo

D. EKG Exercise test

E. Cardiac MRI

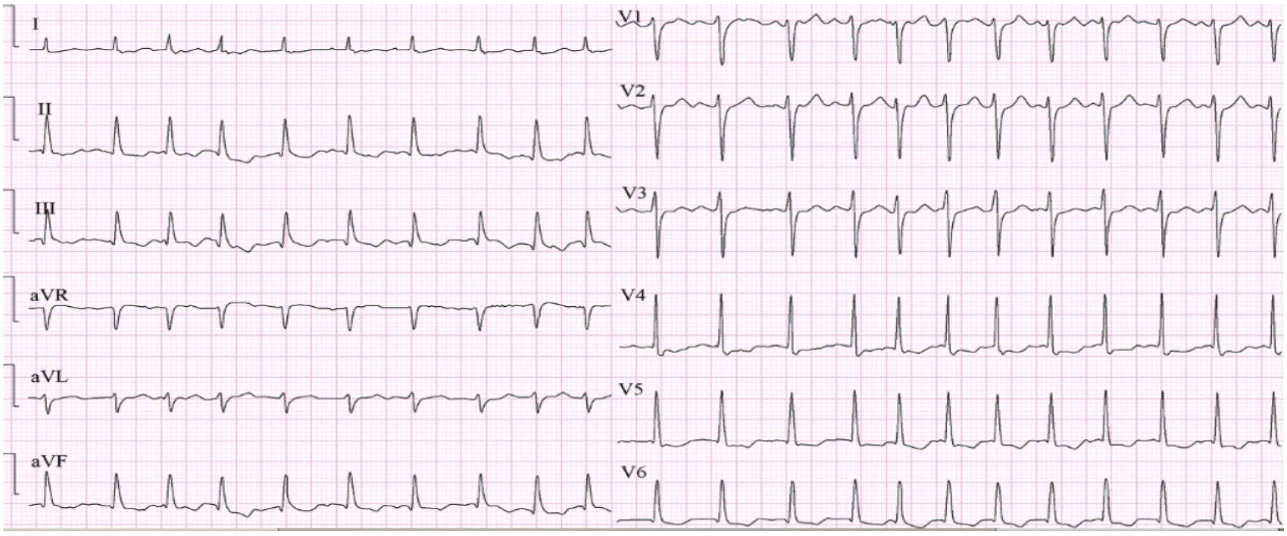

Q5: What did the EKG reveal?

A. Atrial fibrillation
B. Abnormal repolarization
C. Left ventricular hypertrophy
D. No Left ventricular hypertrophy
E. Wolff Parkinson White syndrome

Q6: Considering imaging tests bellow, what tests are necessary for this patient?

A. Holter EKG
B. No other tests
C. Brain scan
D. EMG
E. Thyroid echography

Q7: Among biological tests bellow, what is your prescription?

A. Complete blood count
B. Blood potassium level
C. TSH dosage
D. Urinary potassium level
E. T3 and T4

Q8: Which score do you calculate to evaluate thrombotic risk?

A. CHADS
B. CHA2DS2VASC
C. HASBLED
D. ATRIA
E. HEMORR2HAGES

Q9: What is the CHA2DS2VASC score in this patient?

A. 1
B. 2
C. 3
D. 4
E. 5

Q10: With this score, what is the estimated stroke rate per year of this patient?

A. 1%
B. 2%
C. 3%
D. 4%
E. 5%

Q11: Which score do you calculate to evaluate hemorrhagic risk?

A. CHADS
B. CHA2DS2VASC
C. HASBLED
D. ATRIA
E. HEMORR2HAGES

Q12: What is the hemorrhagic score HASBLED in this patient?

A. 1
B. 2
C. 3
D. 4
E. 5

	Score
Hypertension artérielle non contrôlée (PAS $\geq$ 160mmHg)	1
Dysfonction, rénale ou hépatique	1 ou 2
Accident Vasculaire Cérébral (récent)	1
Maladie à risque hémorragie	1
INR instable ou élevé	1
Âge supérieur à 65 ans	1
Alcoolisme	1
Utilisation de médicaments à action antithrombotique	1
Score maximum possible 9	

Q13: Transthoracic echo is normal. Palpitations started a week ago. What is your final diagnosis?

A. Persistent atrial fibrillation
B. Chronic atrial disease
C. Paroxysmal atrial fibrillation
D. Junctional tachycardia
E. Atrial flutter

Q14: What is your strategy to reduce atrial fibrillation? Several strategies are possible.

A. Beta blocker
B. Flecainide
C. External electric choc after transosophageal echo.
D. External electric choc after 3 weeks of curative anticoagulation
E. Direct external electric choc without weeks of curative anticoagulation

Q15: Which curative anticoagulation do you prescribe? Several strategies are possible.

A. Aspirin
B. Vitamin K antagonist with heparin introduction
C. Vitamin K antagonist without heparin introduction
D. Direct oral anticoagulants without heparin introduction
E. Direct oral anticoagulants with heparin introduction
